# Bitter Taste Receptor Polymorphisms and Human Aging

**DOI:** 10.1371/journal.pone.0045232

**Published:** 2012-11-02

**Authors:** Daniele Campa, Francesco De Rango, Maura Carrai, Paolina Crocco, Alberto Montesanto, Federico Canzian, Giuseppina Rose, Cosmeri Rizzato, Giuseppe Passarino, Roberto Barale

**Affiliations:** 1 Genomic Epidemiology Group, German Cancer Research Center (DKFZ), Heidelberg, Germany; 2 Department of Cell Biology, University of Calabria, Rende, Italy; 3 Department of Biology, University of Pisa, Pisa, Italy; Barnard College, Columbia University, United States of America

## Abstract

Several studies have shown that genetic factors account for 25% of the variation in human life span. On the basis of published molecular, genetic and epidemiological data, we hypothesized that genetic polymorphisms of taste receptors, which modulate food preferences but are also expressed in a number of organs and regulate food absorption processing and metabolism, could modulate the aging process. Using a tagging approach, we investigated the possible associations between longevity and the common genetic variation at the three bitter taste receptor gene clusters on chromosomes 5, 7 and 12 in a population of 941 individuals ranging in age from 20 to 106 years from the South of Italy. We found that one polymorphism, rs978739, situated 212 bp upstream of the *TAS2R16* gene, shows a statistically significant association (p = 0.001) with longevity. In particular, the frequency of A/A homozygotes increases gradually from 35% in subjects aged 20 to 70 up to 55% in centenarians. These data provide suggestive evidence on the possible correlation between human longevity and taste genetics.

## Introduction

Several studies including various genome wide association studies have demonstrated the existence of an important familial and genetic component of longevity [1], [2], [3], [4], [5], [6], [7], [8], [9]. Twin studies have highlighted that approximately 25% of the overall variation in human lifespan can be attributed to genetic factors [10], [11], [12], which becomes more relevant after 60 years of age [13]. Based on the results obtained in model organisms, the research on the genetic component of human longevity has been focused on conserved pathways related to stress response signalling, DNA repair and to the storage and the use of nutrients [14], [15]. Studies on centenarians or long-lived subjects allowed indeed to identify specific genes and genotypes involved in these pathways that influence human lifespan (for reviews see [16], [17], [18], [19]). In particular, the variability and the expression of the genes involved in the storage and the use of the nutrients showed to influence both longevity and the quality of the aging [14], [20].

The importance of nutrients in the aging process is also witnessed by overwhelming epidemiologic evidences that diet and nutrition can affect growth, the development of the body during childhood, the risk of acute and chronic diseases during adulthood, the maintenance of physiological processes and the biological process of aging [21], [22],[23],[24],[25],[26],[27],[28],[29],[30],[31],[32],[33],[34],[35],[36],[37],[38]. In particular, diets rich in vegetables seem to be associated with a significant increase in longevity and wellness [23], [39], [40]. In epidemiological studies it could be difficult to obtain, by elders, reliable information on diet style in their early decades of life, which, likely, significantly influenced later health status. Since there are several indications that bitter taste gene polymorphisms can influence food choice [39], [41], [42], [43], [44], [45], we considered of some interest to investigate the possible association between bitter taste and longevity. On the basis of these molecular, genetic and epidemiological data from the literature we hypothesized that genetic polymorphisms of taste receptors, which modulate food preferences but are also expressed in a number of organs and regulate food absorption and processing, could modulate the aging process.

For example, the *TAS2R38* gene is characterized by three non synonymous coding SNPs (rs713598 – G145C, Ala49Pro; rs1726866 – T785C, Val262Ala; rs10246939 – A886G, Ile296Val) which give rise to several haplotypes [46]. Subjects possessing at least one copy of the PAV haplotype (i.e. the alleles coding for proline at rs713598, alanine at rs1726866 and valine at rs10246939) are significantly more responsive to the bitter tastants PROP, PTC, and chemically similar compounds [47], [48], [49], [50], [51], [52], [53], [54], [55]. Such individuals display the so-called taster phenotype, and are distinct from those who are homozygous for the AVI haplotype and display the so-called non-taster phenotype. Tasters show a reduction in their intake of several vegetables such as cabbage, spinach, lettuce [56], [57]. Given the importance of diet in longevity, genetic variation in taste receptor could directly affect a healthy aging by modulating food preference during life.

On the other hand, new evidence strongly suggests that taste genes play a much broader role in human health. Genes of the *TAS1R*-*TAS2R* gene family express membrane taste receptors in the neuroendocrine cells of several organs of the gastrointestinal system [58]. These cells start the regulation of a variety of relevant functions, including appetite, satiety, the proliferation of epithelial GI cells, secretory activity of the stomach, liver and pancreas, intestine motility, and gall bladder contraction [59]. A strong correlation has been observed between polymorphic variants in various taste receptors and homeostasis of glucose and insulin [60], which in turn are strongly related with longevity [61]. Therefore allelic variants in the taste receptor family may be a link between ageing and glucose homeostasis. Moreover candidate gene studies on various traits have indicated suggestive associations between allelic variants in taste receptor genes and Body Mass Index (BMI) [34], [62], [63], complex diseases such as cancer [64], alcohol consumption [65], smoking [66] and nicotine dependence [67] although these associations did not emerge from genome-wide association studies on BMI and diabetes, at the highly stringent threshold that is typically used in such studies (p∼10^−8^). Recently it has been shown that taste receptors are expressed in several organs and tissues which are not directly implicated in food intake or digestion such as the lung, the airway smooth muscle, the nose and the testis. In the airways they are thought to enhance the response to noxious stimuli, while in the testis they could be involved in spermatogenesis. These findings point to the fact that bitter sensing is just one of the functions performed by this cluster of genes, which could have a central role in the homeostasis of the organisms. Given the possible involvement of the taste genes in a wide spectrum of aspects which are crucial to health and survival such as diet and nutrition or the defense of the organism from aerial and ingested toxic compounds, and due to the fact that the genetic variability within these genes is well documented to have an impact on their function, we addressed the hypothesis that taste receptor genetic variants may have a role in longevity. In particular, using a tagging approach, we investigated the possible association between the common genetic variation at the three bitter taste gene clusters on chromosomes 5, 7 and 12 and longevity in a population of 941 individuals ranging from 60 to 106 years of age from the South of Italy.

**Figure 1:**
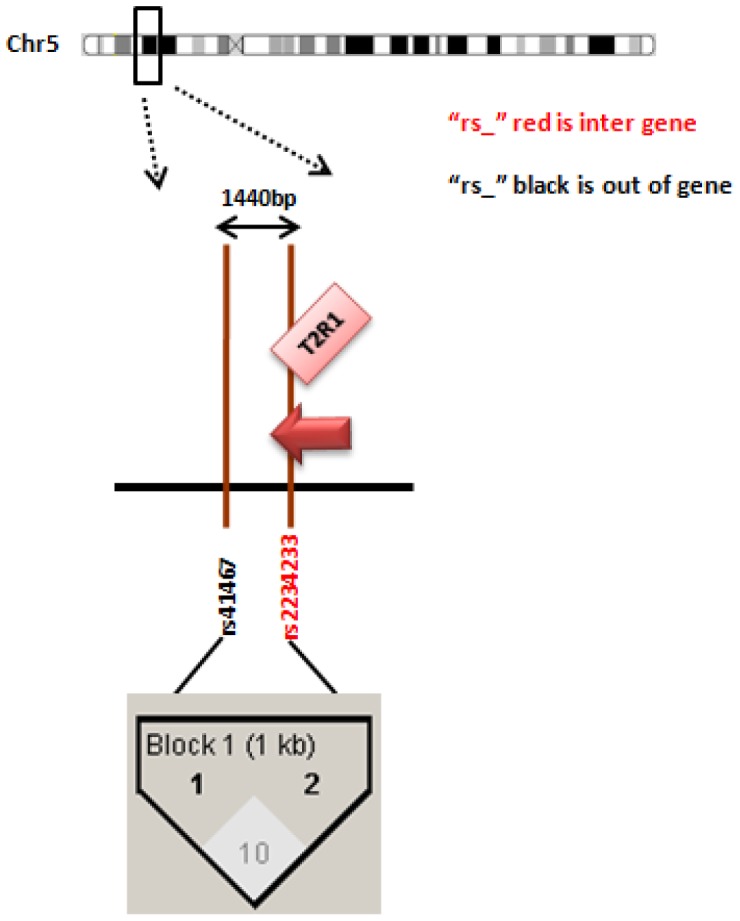
Figure 1 shows the selected polymorphisms in the chromosome 5 region. The value inside each diamond represents the linkage disequilibrium (r^2^) between each SNP. The values inside the black arrows represent the distance between the SNPs. SNPs situated in a coding region are written in red, while SNPs in non coding regions are in black. The red arrows show the direction in which the gene is transcribed.

**Figure 2:**
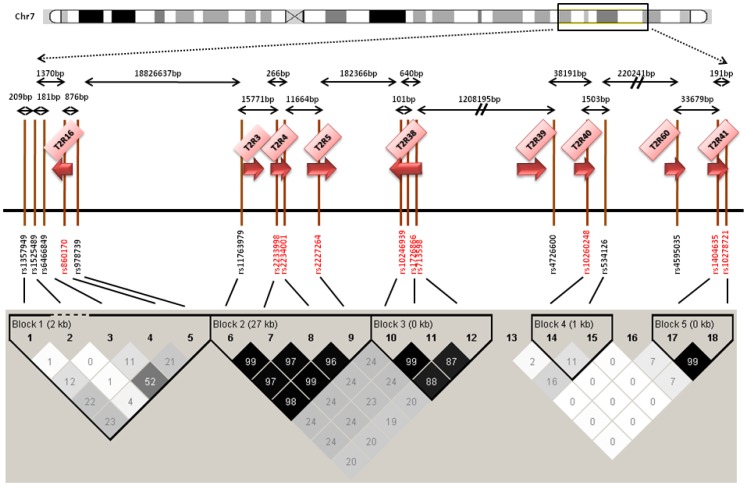
Figure 2 shows the selected polymorphisms in the chromosome 7 region. Symbols are as in Figure 1.

**Figure 3:**
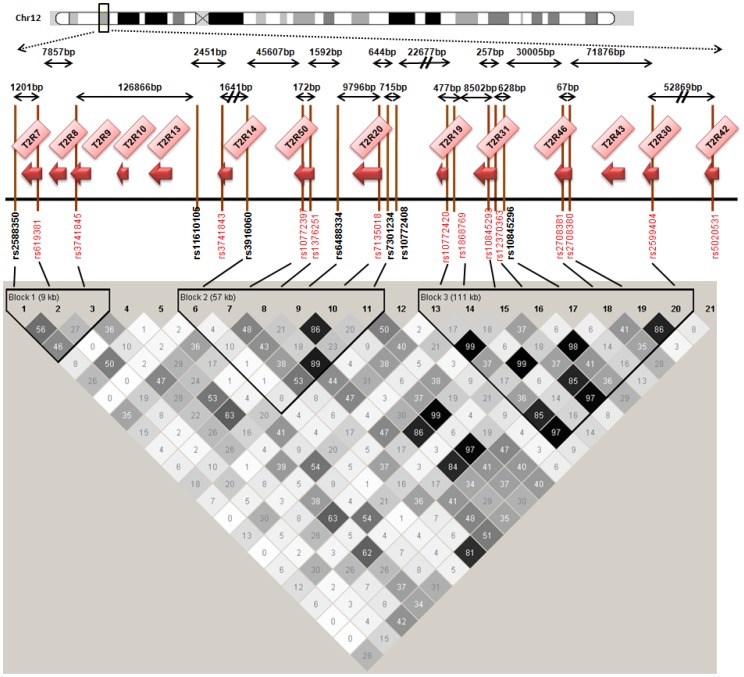
Figure 3 shows the selected polymorphisms in the chromosome 12 region. Symbols are as in Figure 1.

## Materials and Methods

### Ethical Statement

Samples were collected within the framework of several recruitment campaigns carried out for monitoring the quality of aging in Calabria (Southern Italy) from 2002. The recruitment campaigns and subsequent analyses received the approval of the Ethical committee of the University of Calabria. All subjects provided written informed consent for studies on aging carried out by our research group. White blood cells (WBC) from blood buffy coats were used as a source of DNA.

**Table 1:** SNPs in chromosome 7 cluster associated with longevity.

ID_Gene	SNP	≥85 yrs^a^	<85 yrs^a^	OR (95%CI)^b^	P_value_	P_trend_
*T2R16*	rs6466849					
	G/G	233	316	1		0.043
	A/G	86	172	0.69 (0.50–0.94)	0.018	
	A/A	14	22	0.89 (0.44–1.77)	0.730	
	(A/G+A/A)			0.71 (0.53–0.95)	0.023	
*T2R16*	rs860170					
	A/A	139	253	1		0.042
	A/G	153	224	1.25 (0.93–1.67)	0.135	
	G/G	39	46	1.51 (0.94–2.43)	0.090	
	(A/G+G/G)			1.29 (0.98–1.71)	0.069	
*T2R16*	rs978739					
	A/A	185	245	1		**0.004**
	A/G	125	284	0.59 (0.45–0.79)	3.26*10^−4^	
	G/G	30	54	0.76 (0.46–1.23)	0.262	
	(A/G+G/G)			0.62 (0.47–0.81)	**0.001**	
*T2R4*	rs2233998					
	C/C	118	159	1		0.057
	C/T	146	282	0.71 (0.52–0.97)	0.029	
	T/T	78	146	0.74 (0.51–1.06)	0.102	
	(C/T+T/T)			0.72 (0.54–0.96)	0.024	
*T2R5*	rs2227264					
	T/T	114	158	1		0.047
	G/T	148	287	0.72 (0.53–0.99)	0.044	
	G/G	74	146	0.72 (0.50–1.04)	0.081	
	(G/T+G/G)			0.72 (0.54–0.97)	0.029	

a Numbers may not add up to 100% of subjects due to genotyping failure. Data points that were still not filled after this procedure were left blank.

b OR: odds ratio; CI: confidence interval.

### Study Population

Subjects were recruited between 1994 and 2008 in Calabria and included 941 unrelated individuals, of which 348 were very elderly cases (≥85 years, range 85–106 years, mean age 93.82±4.44 years, median age 92) and 593 were non-elderly controls (<85 years, range 20–84 years, mean age 59.17±19.45 years, median age 67 years). We chose 85 as a cut-off point because it has been shown that genetic factors contribute to the variation in human life span minimally before age 60 years and most profoundly from age 85 years onwards [13]. Study participants, their parents, and grandparents were all born in Calabria, as ascertained from population registers.

Younger subjects were contacted through general physicians. Subjects older than 90 years were identified by screening of population registers in different municipalities distributed across the Calabria region. It is important to note that we can exclude the fact that older and younger people in Calabria are of two different ethnicities. Calabria is not subject to immigration from other parts of Italy and all the subjects in the study were of Caucasian origin excluding the possibility that the observed effect is due to ethnicity Eligible subjects were contacted and invited to participate in the study. Written informed consent was obtained from all participants before the enrolment in the study. The health status was ascertained through a medical examination carried out by a geriatrician. Subjects with dementia and/or neurologic disorders were not included. At the time of the visit, peripheral venous blood samples were also obtained.

**Figure 4:**
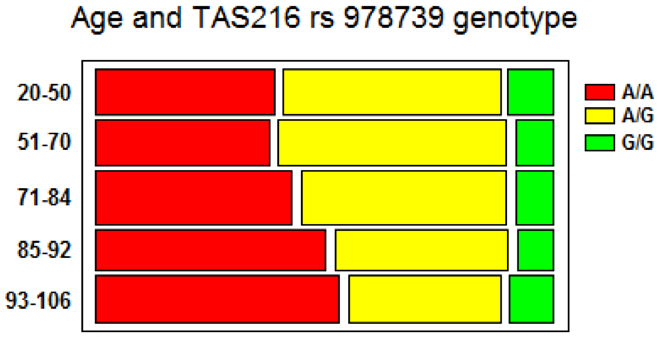
Bar chart of age by genotypes showing the association between the polymorphism of *TAS2R16* rs 978739 and longevity. The A/A genotype frequency is about 40.5% in the young and adult classes (20–50 and 51–70 years). Thereafter the A/A frequency increases through older classes reaching the 55.5% in the oldest (χ^2^ = 17,08, p<0.029).

### Selection of Tagging SNPs

We sought to survey the entire set of common genetic variants in the selected bitter taste receptors situated at chromosome 5, 7 and 12. To this end, we followed a hybrid tagging-functional approach. We used the algorithm described by Carlson and coworkers [68] that was developed to select the maximally informative set of tag SNPs in a candidate gene/candidate region for an association study. The resulting SNPs captured all genetic variability in the three regions of interest. All polymorphisms with minor allele frequency (MAF) ≥5% in Caucasians from the International HapMap Project (version 28, August 2010; http://www.hapmap.org) were included. Tagging SNPs were selected with the use of the Tagger program within Haploview (http://www.broad.mit.edu/mpg/haploview/; http://www.broad.mit.edu/mpg/tagger/) [69], [70], using pairwise tagging with a minimum r^2^ of 0.8. On chromosome 5 we considered a 1440 base-pair region (9628089–9629529) extending from rs41467 to rs2234233 in the *TAS2R1* gene. For genes situated on chromosome 7 and 12 we selected tagging polymorphisms. For the *TAS2R38* gene we selected rs713598, rs1726866 and rs10246939 as tagging SNPs since they are all non-synonymous and functional [51]. The final selection included 41 SNPs belonging to 20 genes. [51]. Figures 1, 2 and 3 show the selected polymorphisms in the chromosome 5, 7 and 12 regions respectively, as well as the distance and the LD between each SNP within the same chromosome. Table S1 shows the genes and SNPs selected in the study, the Hardy-Weinberg equilibrium (HWE) values observed for each SNP in the study, their position in the genome, in the gene, and the amino acidic change specified.

### DNA Extraction and Genotyping

DNA was extracted from blood samples with standard proteinase K digestion followed by phenol/chloroform extraction and ethanol precipitation. The order of DNAs of cases (age≥85) and controls (age<85) was randomized on PCR plates in order to ensure that an equal number of cases and controls could be analyzed simultaneously. All genotyping was carried out using Kaspar (Kbioscience, Heddesdon, UK) or Taqman (Applied Biosystems, Foster City, CA, USA) assays. PCR plates were read on an ABI PRISM 7900HT instrument (Applied Biosystems).

### Haplotype Reconstruction

Haplotype blocks were identified from the genotyping data of this study using SNPtool (http://www.dkfz.de/de/molgen_epidemiology/tools/SNPtool.html) [71] and the Haploview v4.2 software using a MAF of 0.05, an HWE p-value of 0.001 and a call rate of 75% as cut-off values. Individual haplotypes were then statistically inferred using the PHASE v.2.1.1 algorithm, based on a Bayesian approach (http://www.stat.washington.edu/stephens/) [72].

### Statistical Analysis

The frequency distribution of genotypes was examined for the cases and the controls. HWE was tested for each of the SNP by chi-square test. logistic regression for multivariate analyses to assess the main effects of the genetic polymorphism on longevity was used. In these models the genetic data was coded using a co-dominant and a dominant inheritance model using the most common genotype in the controls as the reference category. All analyses were adjusted for gender. For haplotype analysis unconditional logistic regression was used to estimate the “risk of longevity”. The most frequent haplotype was set as reference, while haplotypes with a frequency below 1% were declared as rare and combined in a single category. In order to take into account the large number of tests performed in this study, we calculated for each gene the number of effective independent variables, M_eff_, by use of the SNP Spectral Decomposition approach [73]. We obtained a region-wide M_eff_ value for each chromosomal region and also a study-wide M_eff_ value, by adding up region M_eff_’s.

All analysis were performed using STATGRAPHICS*®* Centurion XVI software (© 2009 by StatPoint Technologies, Inc. www.STATGRAPHICS.com) and STATA software (StataCorp, College Station, TX).

### Gene-gene Interactions

SNP-SNP interactions were tested using nonparametric Multifactor Dimensionality Reduction (MDR, http://www.epistasis.org.) and verified with logistic regression. MDR is a data reduction approach for detecting interactions of multilocus genotypes and discrete environmental factors that are predictive of a discrete outcome. MDR defines a single variable that incorporates information from several loci and/or environmental factors and evaluates its ability to classify and predict outcome risk status (higher or lower) using cross-validation and permutation testing. Detailed information is described elsewhere [74].

## Results

### Genotyping Success Rates and Quality Control

The genotype distributions at all loci were in HWE with non-significant chi square values (p>0.05) (table S1). Random duplicate samples (8%) were also included and concordance of their genotypes was greater than 99%. The average call rate for the SNPs was 97.% (range 90%–100%).

### Main Effects of Genotyped SNPs

The distribution of the genotypes and their odds ratios (ORs) for association with longevity are shown in Table S2. We found that there were five significant associations between the SNPs in the chromosome 7 cluster and longevity at the conventional level of p<0.05. Three were observed in the *TAS2R16* gene (rs6466849, rs860170 and rs978739), one in the *TAS2R4* gene (rs2233998), and one in the *TAS2R5* gene (rs2227264) (shown in Table 1). All the variant alleles were associated with a decreased probability to attain longevity (ORs<1).

To take into account multiple testing, we calculated for each region the number of effective independent variables (M_eff_). The M_eff_ values for the regions on chromosome 5, chromosome 7 and chromosome 12 were 2, 11 and 8, respectively. The experiment-wise significance threshold was therefore set at 0.05/(2+11+8) = 0.0024. After correction for multiple testing, only the carriers of the G allele of rs978739 variation showed a statistically significant association.

Haplotype analysis was performed for each LD block in the study. None of the haplotypes showed a study-wise statistically significant association with longevity. However, the haplotype (rs1357949–rs6466849–rs860170–rs978739: T_A_A_G) of the *TAS2R16* gene showed a suggestive association with longevity (OR = 0.74; 95% CI 0.56–0.98; *p* = 0.033). Moreover the haplotype rs2588350 (TAS2R7)-rs619381(TAS2R9)-rs3741845(TAS2R9): G_G_T showed an association with increased longevity at the conventional 0.05 P-value (OR = 1.35; 95% CI 1.02–1.78; *p* = 0.037). Tables S3, S4, S5, S6, S7, S8, S9, S10, and S11 show the distribution of all the haplotypes and their associations with longevity. We also analyzed all the possible pair-wise interactions between SNPs using the MDR method. No interactions between SNPs emerged with a study-wise significant *p*-value.

## Discussion

In the present study, we have investigated possible associations between longevity and 41 SNPs in 20 candidate genes involved in bitter taste perception. Our intensive SNP tagging approach along with the analyses of haplotypes provides a close to exhaustive analysis of the possible associations of longevity with the known common polymorphic variants at these bitter taste receptor loci.

We found that five polymorphisms were associated with longevity: three in the *TAS2R16* gene (rs6466849, rs860170 and rs978739), one in the *TAS2R4* gene (rs2233998) and two in the *TAS2R5* (rs2227264). rs6466849 and rs978739 share a modest degree of LD (r^2^ = 0.52) indicating that they might not be statistically independent findings, while rs2233998 and rs2227264 are in complete LD (r^2^ = 0.99) and therefore represent the same association. Two haplotypes, (rs1357949–rs6466849–rs860170–rs978739: T_A_A_G) of the *TAS2R16* gene and rs2588350 (*TAS2R7*)-rs619381(*TAS2R9*)-rs3741845(*TAS2R9*): G_G_T showed a suggestive association with longevity. After correction for multiple testing only one polymorphism, rs978739 showed a statistically significant association with longevity (p = 0.001). In particular, the frequency of homozygotes A/A increases gradually from 35% in the subjects aged 20 to 70 up to 55% in centenarians. A distribution of the genotypes through various age strata is given in Figure 4.

The *TAS2R16* gene is one of the most studied genes in the bitter taste receptor family and interestingly it has been shown that it underwent recent selective pressure [75]. It mediates the detection of salicin and other naturally occurring bitter compounds such as diphenidol, sodium benzoate, amygdalin, arbutin, helicin, D-salicin, sinigrin, and phenyl beta-D-glucopyranoside [76], [77]. Several of these compounds have been reported to have a pharmacologic effect and to be present in human food. For example, arbutin is present in pears, bearberries and wheat, and has been reported to be a strong inhibitor of bladder cancer proliferation [78]. Amygdalin, also known as Vitamin B17, is found in several fruit seeds and has been reported to have both apoptotic activity and to inhibit cell cycle genes [79] although its real effect on cancer remains controversial [80]. Sinigrin is found in plants of the *Brassicaceae* family such as broccoli, brussels sprouts, and the seeds of black mustard. It has been proposed to have a preventive effect on colorectal cancer and to inhibit bladder cancer [81]. The bark and leaf of willow species contain the prodrug salicin; following absorption salicin is metabolized into various salicylate derivatives [82]. Salicin has effects similar to aspirin (acetylsalicylic acid) on analgesia and as an anti-inflammatory agent [82]. These reports point to a role for the TAS2R16 receptor in recognizing beneficial molecules with which the organism interacts during life. One can speculate that an impaired function of the receptor might affect the efficacy of the various compounds and that this could lead on the long term to a disadvantage for the organism.

Polymorphic variants in TAS2R16 confer differential response *in vitro* via functional changes in the receptor [83] and have been suggested to influence the sensations, liking, or intake of common beverages that contain phytochemicals and other pharmacologically active ingredients linked to chronic diseases [84]. Moreover the functional polymorphism K172N (rs846664) appears to be a risk factor for alcohol intake [85] and dependence [86]. This variant is very rare in Caucasian populations and therefore its genotyping was not attempted in this sample set. *TAS2R16* genetic variants have also been associated with the development of nicotine dependence in African Americans [67].

These observations point to a role of variation in the TAS2R16 receptor in recognizing and therefore modulating the effect of both beneficial and harmful molecules with which the organism interacts during life. It is possible that the fine tuning of the receptor function due to the genetic polymorphisms along with the environment may modulate how many beneficial and how many harmful compounds are recognized by the receptor throughout the life span and that this could, in the long term, modify the chances to reach very old ages. However there is also another possible, even though highly speculative, explanation of the involvement of TAS2R16 genetic variability in healthy aging. Numerous recent reports investigated non-gustatory actions of taste receptors. They have been shown to be expressed in a plethora of tissues such as the respiratory system where they affect respiratory functions in response to noxious stimuli [87], and the gastrointestinal tract where they are suspected to regulate the activation of metabolic and digestive functions [87]. Recently it has been shown that taste receptors are expressed also in the testis in mouse, where they can be involved in spermatogenesis [88]. The emerging picture is therefore that taste receptors could behave as pleiotropic genes, whose products are used by various cells, or have signaling function on various targets not linked one to the other. Probably the bitter sensing is just one of the functions performed by this cluster of genes, which could have a central role in the homeostasis of the organisms. Therefore their genetic variations can affect profoundly various traits, including longevity, in a way that we are just beginning to understand [89].

## Supporting Information

Table S1 **Table S1 Shows the genes and SNPs selected in the study, the Hardy-Weinberg equilibrium (HWE) values observed for each SNP in the study, their position in the genome, in the gene, and the amino acidic change specified.**
(DOC)

Table S2 **Logistic regression analysis for taste SNPs in long lived subjects.**
(DOCX)

Table S3 **Logistic regression analysis for haplotypes of** ***T2R1*** **gene in long lived subjects.**
(DOCX)

Table S4 **Logistic regression analysis for haplotypes of** ***T2R3-T2R4-T2R5-*** **genes in long lived subjects.**
(DOCX)

Table S5 **Logistic regression analysis for haplotypes of** ***TAS2R16*** **gene in long lived subjects.**
(DOCX)

Table S6 **Logistic regression analysis for haplotypes of** ***T2R38*** **gene in long lived subjects.**
(DOCX)

Table S7 **Logistic regression analysis for haplotypes of** ***T2R40*** **gene in long lived subjects.**
(DOCX)

Table S8 **Logistic regression analysis for haplotypes of** ***T2R41*** **in long lived subjects.**
(DOCX)

Table S9 **Logistic regression analysis for haplotypes of** ***T2R7 -T2R9*** **genes in long lived subjects.**
(DOCX)

Table S10 **Logistic regression analysis for haplotypes of** ***T2R14-T2R50-T2R20*** **genes in long lived subjects.**
(DOCX)

Table S11 **Logistic regression analysis for haplotypes of** ***T2R19-T2R31-T2R46-T2R30*** **genes in long lived subjects.**
(DOCX)
