# Supplementary material for: Bitter Taste Receptor Polymorphisms and Human Aging
Source: PLoS One. 2012 Nov 2;7(11):e45232. doi: 10.1371/journal.pone.0045232 (PMC3487725; doi:10.1371/journal.pone.0045232)
Supplement: Table S1 — Shows the genes and SNPs selected in the study, the Hardy-Weinberg equilibrium (HWE) values observed for each SNP in the study, their position in the genome, in the gene, and the amino acidic change specified. (DOC) [file pone.0045232.s001.doc]

**Supplementary table S1**

|  | **ID_Gene** | **rs_** | **SNP** | **MAF** | | **H-W p-value** | **Position bp^a^** | **Function^a^** |
| --- | --- | --- | --- | --- | --- | --- | --- | --- |
|  |  |  |  |  |  |  |  |  |
| **Chromosome 5** |  |  |  |  |  |  |  |  |
|  |  |  |  |  |  |  |  |  |
|  | *TAS2R1* | rs41467 | G/T | f(T) | 0.39 | 0.485 | 9628089 | non-coding |
|  |  | rs2234233 | C/T | f(T) | 0.14 | 0.947 | 9629529 | R206W |
| **Chromosome 7** |  |  |  |  |  |  |  |  |
|  |  |  |  |  |  |  |  |  |
|  | *TAS2R16* | rs1357949 | T/C | f(C) | 0.33 | 0.423 | 122633264 | non-coding |
|  |  | rs6466849 | G/A | f(A) | 0.19 | 0.661 | 122633654 | non-coding |
|  |  | rs860170 | A/G | f(G) | 0.32 | 0.959 | 122635024 | missense H222R |
|  |  | rs978739 | A/G | f(G) | 0.31 | 0.222 | 122635900 | non-coding |
|  | *TAS2R3* | rs11763979 | T/G | f(G) | 0.47 | 0.081 | 141462537 | non-coding |
|  | *TAS2R4* | rs2233998 | C/T | f(T) | 0.48 | 0.087 | 141478308 | missense F7S |
|  |  | rs2234001 | C/G | f(G) | 0.47 | 0.070 | 141478574 | missense V96L |
|  | *TAS2R5* | rs2227264 | T/G | f(G) | 0.47 | 0.178 | 141490238 | missense S26I |
|  | *TAS2R38* | rs10246939 | C/T | f(T) | 0.47 | 0.766 | 141672604 | missense I296V |
|  |  | rs1726866 | G/A | f(A) | 0.47 | 0.766 | 141672705 | missense V262A |
|  |  | rs713598 | C/G | f(G) | 0.50 | 0.766 | 141673345 | missense A49P |
|  | *TAS2R39* | rs4726600 | G/A | f(A) | 0.18 | 0.822 | 142881540 | non-coding |
|  | *TAS2R40* | rs10260248 | C/A | f(A) | 0.08 | 0.391 | 142919731 | missense S187Y |
|  |  | rs534126 | C/T | f(T) | 0.44 | 0.791 | 142921234 | non-coding |
|  | *TAS2R60* | rs4595035 | C/T | f(T) | 0.38 | 0.397 | 143141475 | synonymous R310R |
|  | *TAS2R41* | rs1404635 | G/A | f(A) | 0.21 | 0.843 | 143175154 | synonymous T63T |
|  |  | rs10278721 | C/T | f(T) | 0.21 | 0.955 | 143175345 | missense P127L |
| **Chromosome 12** |  |  |  |  |  |  |  |  |
|  |  |  |  |  |  |  |  |  |
|  | *TAS2R7* | rs2588350 | G/A | f(A) | 0.20 | 0.558 | 10953057 | non-coding downstream |
|  |  | rs619381 | G/A | f(A) | 0.13 | 0.739 | 10954258 | missense M304I |
|  | *TAS2R9* | rs3741845 | T/C | f(C) | 0.34 | 0.366 | 10962115 | missense V187A |
|  | *TAS2R14* | rs11610105 | G/A | f(A) | 0.22 | 0.851 | 11088981 | non-coding |
|  |  | rs3741843 | T/C | f(C) | 0.17 | 0.664 | 11091432 | synonymous R125R |
|  |  | rs3916060 | T/C | f(C) | 0.09 | 0.414 | 11093073 | non-coding |
|  | *TAS2R50* | rs10772397 | A/G | f(G) | 0.34 | 0.884 | 11138680 | synonymous P259P |
|  |  | rs1376251 | C/T | f(T) | 0.48 | 0.288 | 11138852 | missense C203Y |
|  |  | rs6488334 | G/A | f(A) | 0.18 | 0.187 | 11140444 | non-coding |
|  | *TAS2R49* | rs7135018 | C/T | f(T) | 0.17 | 0.239 | 11150240 | missense K79E |
|  |  | rs7301234 | T/C | f(C) | 0.49 | 0.357 | 11150884 | non-coding |
|  |  | rs10772408 | G/A | f(A) | 0.32 | 0.280 | 11151599 | non-coding |
|  | *TAS2R48* | rs10772420 | C/T | f(T) | 0.34 | 0.174 | 11174276 | missense R299C |
|  | *TAS2R44* | rs10845293 | C/T | f(T) | 0.34 | 0.682 | 11183255 | missense A227V |
|  |  | rs12370363 | T/C | f(C) | 0.16 | 0.151 | 11183512 | synonymous A141A |
|  |  | rs10845296 | T/C | f(C) | 0.25 | 0.622 | 11184140 | non-coding |
|  | *TAS2R46* | rs2708381 | G/A | f(A) | 0.16 | 0.232 | 11214145 | nonsense |
|  |  | rs2708380 | T/A | f(A) | 0.32 | 0.161 | 11214212 | missense L228M |
|  | *TAS2R47* | rs2599404 | T/G | f(G) | 0.35 | 0.584 | 11286088 | missense F252L |
|  | *TAS2R42* | rs5020531 | C/T | f(T) | 0.35 | 0.453 | 11338957 | missense F196S |
|  |  |  |  |  |  |  |  |  |

**^a^** Position of SNP on chromosomes, in base pairs and their function reffered to: http://www.ncbi.nlm.nih.gov/projects/SNP/)
