# Supplementary material for: Bitter Taste Receptor Polymorphisms and Human Aging
Source: PLoS One. 2012 Nov 2;7(11):e45232. doi: 10.1371/journal.pone.0045232 (PMC3487725; doi:10.1371/journal.pone.0045232)
Supplement: Table S2 — Logistic regression analysis for taste SNPs in long lived subjects. (DOCX) [file pone.0045232.s002.docx]

**Supplementary table S2. Logistic Analysis for taste SNPs in long lived subjects**

|  | **ID_Gene** | **SNP** | **≥85 yrs^a^** | **<85 yrs^a^** | **OR (95%CI)^b^** | **P_value_** | **P_trend_** |
| --- | --- | --- | --- | --- | --- | --- | --- |
| **Chromosome5** |  |  |  |  |  |  |  |
|  | T2R1 | rs41467 |  |  |  |  |  |
|  |  | G/G | 128 | 220 | 1 |  | 0.368 |
|  |  | G/T | 164 | 261 | 1.09 (0.82-1.47) | 0.548 |  |
|  |  | T/T | 43 | 98 | 0.76 (0.50-1.16) | 0.208 |  |
|  |  | (G/T+T/T) |  |  | 1.00 (0.76-1.33) | 0.980 |  |
|  | T2R1 | rs2234233 |  |  |  |  |  |
|  |  | C/C | 243 | 433 | 1 |  | 0.948 |
|  |  | C/T | 81 | 143 | 1.00 (0.73-1.37) | 0.995 |  |
|  |  | T/T | 6 | 12 | 0.88 (0.32-2.37) | 0.794 |  |
|  |  | (C/T+T/T) |  |  | 0.99 (0.73-1.34) | 0.946 |  |
| **Chromosome7** |  |  |  |  |  |  |  |
|  | T2R16 | rs1357949 |  |  |  |  |  |
|  |  | T/T | 134 | 253 | 1 |  | 0.861 |
|  |  | C/T | 120 | 245 | 0.91 (0.67-1.23) | 0.537 |  |
|  |  | C/C | 34 | 63 | 1.02 (0.64-1.63) | 0.935 |  |
|  |  | (C/T+C/C) |  |  | 0.93 (0.70-1.24) | 0.610 |  |
|  | T2R16 | rs6466849 |  |  |  |  |  |
|  |  | G/G | 233 | 316 | 1 |  | 0.043 |
|  |  | A/G | 86 | 172 | 0.69 (0.50-0.94) | 0.018 |  |
|  |  | A/A | 14 | 22 | 0.89 (0.44-1.77) | 0.730 |  |
|  |  | (A/G+A/A) |  |  | 0.71 (0.53-0.95) | 0.023 |  |
|  | T2R16 | rs860170 |  |  |  |  |  |
|  |  | A/A | 139 | 253 | 1 |  | 0.042 |
|  |  | A/G | 153 | 224 | 1.25 (0.93-1.67) | 0.135 |  |
|  |  | G/G | 39 | 46 | 1.51 (0.94-2.43) | 0.090 |  |
|  |  | (A/G+G/G) |  |  | 1.29 (0.98-1.71) | 0.069 |  |
|  | T2R16 | rs978739 |  |  |  |  |  |
|  |  | A/A | 185 | 245 | 1 |  | **0.004** |
|  |  | A/G | 125 | 284 | 0.59 (0.45-0.79) | **3.26*10^-4^** |  |
|  |  | G/G | 30 | 54 | 0.76 (0.46-1.23) | 0.262 |  |
|  |  | (A/G+G/G) |  |  | 0.62 (0.47-0.81) | **0.001** |  |
|  | T2R3 | rs11763979 |  |  |  |  |  |
|  |  | G/G | 110 | 161 | 1 |  | 0.116 |
|  |  | G/T | 145 | 282 | 0.76 (0.56-1.05) | 0.093 |  |
|  |  | G/G | 75 | 145 | 0.77 (0.53-1.12) | 0.176 |  |
|  |  | (G/T+G/G) |  |  | 0.77 (0.57-1.03) | 0.075 |  |
|  | T2R4 | rs2233998 |  |  |  |  |  |
|  |  | C/C | 118 | 159 | 1 |  | 0.057 |
|  |  | C/T | 146 | 282 | 0.71 (0.52-0.97) | 0.029 |  |
|  |  | T/T | 78 | 146 | 0.74 (0.51-1.06) | 0.102 |  |
|  |  | (C/T+T/T) |  |  | 0.72 (0.54-0.96) | 0.024 |  |
|  | T2R4 | rs2234001 |  |  |  |  |  |
|  |  | C/C | 114 | 165 | 1 |  | 0.097 |
|  |  | C/G | 150 | 279 | 0.79 (0.58-1.08) | 0.139 |  |
|  |  | G/G | 74 | 144 | 0.76 (0.53-1.11) | 0.153 |  |
|  |  | (C/G+G/G) |  |  | 0.78 (0.58-1.04) | 0.095 |  |
|  | T2R5 | rs2227264 |  |  |  |  |  |
|  |  | T/T | 114 | 158 | 1 |  | 0.047 |
|  |  | G/T | 148 | 287 | 0.72 (0.53-0.99) | 0.044 |  |
|  |  | G/G | 74 | 146 | 0.72 (0.50-1.04) | 0.081 |  |
|  |  |  |  |  |  |  |  |
|  |  | (G/T+G/G) |  |  | 0.72 (0.54-0.97) | 0.029 |  |
|  | T2R38 | rs10246939 |  |  |  |  |  |
|  |  | C/C | 106 | 161 | 1 |  | 0.150 |
|  |  | C/T | 173 | 296 | 0.89 (0.65-1.21) | 0.441 |  |
|  |  | T/T | 66 | 133 | 0.75 (0.51-1.10) | 0.140 |  |
|  |  | (C/T+T/T) |  |  | 0.84 (0.63-1.13) | 0.252 |  |
|  | T2R38 | rs1726866 |  |  |  |  |  |
|  |  | G/G | 104 | 161 | 1 |  | 0.182 |
|  |  | A/G | 172 | 296 | 0.89 (0.65-1.22) | 0.476 |  |
|  |  | A/A | 66 | 133 | 0.76 (0.52-1.12) | 0.164 |  |
|  |  | (A/G+A/A) |  |  | 0.85 (0.63-1.14) | 0.285 |  |
|  | T2R38 | rs713598 |  |  |  |  |  |
|  |  | G/G | 89 | 145 | 1 |  | 0.512 |
|  |  | C/G | 173 | 295 | 0.94 (0.68-1.31) | 0.728 |  |
|  |  | C/C | 80 | 148 | 0.88 (0.60-1.28) | 0.501 |  |
|  |  | (C/G+C/C) |  |  | 0.92 (0.68-1.25) | 0.603 |  |
|  | T2R39 | rs4726600 |  |  |  |  |  |
|  |  | G/G | 235 | 400 | 1 |  | 0.683 |
|  |  | A/G | 91 | 172 | 0.91 (0.68-1.25) | 0.531 |  |
|  |  | A/A | 12 | 19 | 1.08 (0.51-2.26) | 0.845 |  |
|  |  | (A/G+A/A) |  |  | 0.92 (0.69-1.24) | 0.597 |  |
|  | T2R40 | rs10260248 |  |  |  |  |  |
|  |  | C/C | 287 | 506 | 1 |  | 0.368 |
|  |  | A/C | 53 | 83 | 1.12 (0.77-1.63) | 0.557 |  |
|  |  | A/A | 4 | 4 | 1.70 (0.42-6.89) | 0.457 |  |
|  |  | (A/C+A/A) |  |  | 1.15(0.80-1.65) | 0.464 |  |
|  | T2R40 | rs534126 |  |  |  |  |  |
|  |  | C/C | 95 | 197 | 1 |  | 0.244 |
|  |  | C/T | 172 | 278 | 1.30 (0.95-1.78) | 0.095 |  |
|  |  | T/T | 68 | 116 | 1.22 (0.83-1.80) | 0.310 |  |
|  |  | (C/T+T/T) |  |  | 1.28 (0.95-1.72) | 0.100 |  |
|  | T2R60 | rs4595035 |  |  |  |  |  |
|  |  | C/C | 124 | 236 | 1 |  | 0.659 |
|  |  | C/T | 160 | 249 | 1.24 (0.92-1.66) | 0.155 |  |
|  |  | T/T | 47 | 90 | 1.02 (0.67-1.54) | 0.938 |  |
|  |  | (C/T+T/T) |  |  | 1.18 (0.89-1.56) | 0.244 |  |
|  | T2R41 | rs1404635 |  |  |  |  |  |
|  |  | G/G | 204 | 364 | 1 |  | 0.648 |
|  |  | A/G | 109 | 200 | 0.95 (0.71-1.27) | 0.743 |  |
|  |  | A/A | 19 | 25 | 1.43 (0.76-2.68) | 0.263 |  |
|  |  | (A/G+A/A) |  |  | 1.00 (0.76-1.32) | 0.982 |  |
|  | T2R41 | rs10278721 |  |  |  |  |  |
|  |  | C/C | 204 | 363 | 1 |  | 0.626 |
|  |  | C/T | 108 | 201 | 0.93 (0.70-1.25) | 0.648 |  |
|  |  | T/T | 20 | 25 | 1.48 (0.80-2.76) | 0.211 |  |
|  |  | (C/T+T/T) |  |  | 0.99 (0.75-1.31) | 0.958 |  |
| **Chromosome12** |  |  |  |  |  |  |  |
|  | T2R7 | rs2588350 |  |  |  |  |  |
|  |  | G/G | 214 | 384 | 1 |  | 0.989 |
|  |  | A/G | 106 | 181 | 1.08 (0.80-1.44) | 0.623 |  |
|  |  | A/A | 13 | 27 | 0.89 (0.45-1.77) | 0.739 |  |
|  |  | (A/G+A/A) |  |  | 1.05 (0.79-1.40) | 0.723 |  |
|  | T2R7 | rs619381 |  |  |  |  |  |
|  |  | G/G | 264 | 445 | 1 |  | 0.558 |
|  |  | A/G | 73 | 134 | 0.94 (0.68-1.30) | 0.713 |  |
|  |  | A/A | 5 | 10 | 0.86 (0.29-2.55) | 0.787 |  |
|  |  | (A/G+A/A) |  |  | 0.94 (0.68-1.28) | 0.678 |  |
|  | T2R9 | rs3741845 |  |  |  |  |  |
|  |  | C/C | 146 | 240 | 1 |  | 0.414 |
|  |  | C/T | 156 | 226 | 1.18 (0.88-1.57) | 0.277 |  |
|  |  | T/T | 45 | 65 | 1.15 (0.75-1.77) | 0.528 |  |
|  |  | (C/T+T/T) |  |  | 1.17 (0.89-1.54) | 0.263 |  |
|  | T2R14 | rs11610105 |  |  |  |  |  |
|  |  | G/G | 205 | 356 | 1 |  | 0.392 |
|  |  | A/G | 117 | 211 | 0.98 (0.74-1.30) | 0.890 |  |
|  |  | A/A | 23 | 24 | 1.69 (0.93-3.07) | 0.088 |  |
|  |  | (A/G+A/A) |  |  | 1.05 (0.80-1.38) | 0.713 |  |
|  | T2R14 | rs3741843 |  |  |  |  |  |
|  |  | T/T | 248 | 398 | 1 |  | 0.215 |
|  |  | C/T | 90 | 169 | 0.87 (0.64-1.17) | 0.355 |  |
|  |  | C/C | 10 | 22 | 0.74 (0.34-1.58) | 0.433 |  |
|  |  | (C/T+C/C) |  |  | 0.85 (0.64-1.14) | 0.279 |  |
|  | T2R14 | rs3916060 |  |  |  |  |  |
|  |  | T/T | 284 | 494 | 1 |  | 0.666 |
|  |  | C/T | 60 | 87 | 1.18 (0.82-1.69) | 0.367 |  |
|  |  | C/C | 3 | 9 | 0.56 (0.15-2.11) | 0.394 |  |
|  |  | (C/T+C/C) |  |  | 1.12 (0.79-1.60) | 0.518 |  |
|  | T2R50 | rs10772397 |  |  |  |  |  |
|  |  | A/A | 152 | 248 | 1 |  | 0.434 |
|  |  | A/G | 145 | 271 | 0.90 (0.67-1.20) | 0.464 |  |
|  |  | G/G | 38 | 69 | 0.92 (0.59-1.44) | 0.730 |  |
|  |  | (A/G+G/G) |  |  | 0.90 (0.69-1.19) | 0.465 |  |
|  | T2R50 | rs1376251 |  |  |  |  |  |
|  |  | C/C | 87 | 161 | 1 |  | 0.874 |
|  |  | C/T | 165 | 267 | 1.12 (0.81-1.55) | 0.499 |  |
|  |  | T/T | 78 | 141 | 0.99 (0.68-1.45) | 0.970 |  |
|  |  | (C/T+T/T) |  |  | 1.08 (0.79-1.46) | 0.642 |  |
|  | T2R50 | rs6488334 |  |  |  |  |  |
|  |  | G/G | 220 | 393 | 1 |  | 0.585 |
|  |  | A/G | 97 | 166 | 1.04 (0.77-1.41) | 0.780 |  |
|  |  | A/A | 15 | 22 | 1.28 (0.65-2.53) | 0.474 |  |
|  |  | (A/G+A/A) |  |  | 1.07 (0.80-1.43) | 0.640 |  |
|  | T2R49 | rs7135018 |  |  |  |  |  |
|  |  | C/C | 238 | 416 | 1 |  | 0.574 |
|  |  | C/T | 93 | 156 | 1.05 (0.77-1.42) | 0.767 |  |
|  |  | T/T | 13 | 18 | 1.29 (0.62-2.70) | 0.490 |  |
|  |  | (C/T+T/T) |  |  | 1.07 (0.80-1.43) | 0.638 |  |
|  | T2R49 | rs7301234 |  |  |  |  |  |
|  |  | T/T | 87 | 159 | 1 |  | 0.947 |
|  |  | C/T | 170 | 278 | 1.13 (0.82-1.56) | 0.461 |  |
|  |  | C/C | 79 | 147 | 1.01 (0.69-1.47) | 0.974 |  |
|  |  | (C/T+C/C) |  |  | 1.09 (0.80-1.48) | 0.590 |  |
|  | T2R49 | rs10772408 |  |  |  |  |  |
|  |  | G/G | 157 | 271 | 1 |  | 0.311 |
|  |  | A/G | 148 | 241 | 1.07 (0.80-1.42) | 0.646 |  |
|  |  | A/A | 28 | 72 | 0.68 (0.42-1.11) | 0.122 |  |
|  |  | (A/G+A/A) |  |  | 0.98 (0.75-1.29) | 0.892 |  |
|  | T2R48 | rs10772420 |  |  |  |  |  |
|  |  | C/C | 140 | 243 | 1 |  | 0.591 |
|  |  | C/T | 131 | 241 | 0.94 (0.69-1.26) | 0.661 |  |
|  |  | T/T | 45 | 64 | 1.23 (0.80-1.90) | 0.349 |  |
|  |  | (C/T+T/T) |  |  | 1.00 (0.75-1.32) | 0.984 |  |
|  | T2R44 | rs10845293 |  |  |  |  |  |
|  |  | C/C | 143 | 253 | 1 |  | 0.374 |
|  |  | T/C | 146 | 261 | 0.99 (0.74-1.32) | 0.920 |  |
|  |  | T/T | 47 | 64 | 1.30 (0.85-2.00) | 0.229 |  |
|  |  | (T/C+T/T) |  |  | 1.05 (0.80-1.38) | 0.738 |  |
|  | T2R44 | rs12370363 |  |  |  |  |  |
|  |  | T/T | 240 | 411 | 1 |  | 0.641 |
|  |  | T/C | 90 | 154 | 1.01 (0.74-1.37) | 0.969 |  |
|  |  | C/C | 14 | 18 | 1.36 (0.66-2.79) | 0.402 |  |
|  |  | (T/C+C/C) |  |  | 1.04(0.78-1.40) | 0.779 |  |
|  | T2R44 | rs10845296 |  |  |  |  |  |
|  |  | T/T | 190 | 317 | 1 |  | 0.245 |
|  |  | T/C | 117 | 232 | 0.85 (0.64-1.14) | 0.280 |  |
|  |  | C/C | 19 | 38 | 0.84 (0.47-1.50) | 0.556 |  |
|  |  | (T/C+C/C) |  |  | 0.85 (0.65-1.12) | 0.252 |  |
|  | T2R46 | rs2708381 |  |  |  |  |  |
|  |  | G/G | 239 | 417 | 1 |  | 0.380 |
|  |  | A/G | 90 | 154 | 1.03 (0.76-1.40) | 0.835 |  |
|  |  | A/A | 15 | 16 | 1.67 (0.81-3.44) | 0.168 |  |
|  |  | (A/G+A/A) |  |  | 1.09 (0.82-1.46) | 0.553 |  |
|  | T2R46 | rs2708380 |  |  |  |  |  |
|  |  | T/T | 151 | 286 | 1 |  | 0.362 |
|  |  | T/A | 139 | 236 | 1.11 (0.83-1.48) | 0.472 |  |
|  |  | A/A | 40 | 64 | 1.19 (0.77-1.86) | 0.434 |  |
|  |  | (T/A+A/A) |  |  | 1.13 (0.86-1.48) | 0.381 |  |
|  | T2R47 | rs2599404 |  |  |  |  |  |
|  |  | T/T | 141 | 260 | 1 |  | 0.311 |
|  |  | G/T | 151 | 260 | 1.07 (0.80-1.43) | 0.639 |  |
|  |  | G/G | 47 | 69 | 1.27 (0.83-1.94) | 0.271 |  |
|  |  | (G/T+G/G) |  |  | 1.11 (0.85-1.46) | 0.440 |  |
|  | T2R42 | rs5020531 |  |  |  |  |  |
|  |  | C/C | 150 | 234 | 1 |  | 0.389 |
|  |  | C/T | 153 | 282 | 0.87 (0.65-1.15) | 0.330 |  |
|  |  | T/T | 41 | 71 | 0.95 (0.61-1.47) | 0.819 |  |
|  |  | (C/T+T/T) |  |  | 0.88 (0.67-1.16) | 0.372 |  |
|  |  |  |  |  |  |  |  |

**^a^** Numbers may not add up to 100% of subjects due to genotyping failure. Data points that were still not filled after this procedure were left blank.

**^b^** OR: odds ratio; CI: confidence interval
