# Supplementary material for: Bitter Taste Receptor Polymorphisms and Human Aging
Source: PLoS One. 2012 Nov 2;7(11):e45232. doi: 10.1371/journal.pone.0045232 (PMC3487725; doi:10.1371/journal.pone.0045232)
Supplement: Table S3 — Logistic regression analysis for haplotypes of T2R1 gene in long lived subjects. (DOCX) [file pone.0045232.s003.docx]

**Supplementary table S3: Logistic Analysis for Haplotypes of *T2R1* gene in long lived subjects**

**Chromosome 5**

|  | **rs41467** | **rs2234233** |  |  |  |  |
| --- | --- | --- | --- | --- | --- | --- |
| **Haplotypes** | ***T2R1*** | ***T2R1*** | **≥85yrs^a^** | **<85yrs^a^** | **OR (95% CI)^b^** | **P_value_** |
| Haplotype1: | G | C | 309 | 532 | 1 |  |
| Haplotype2: | T | C | 243 | 454 | 0.93 (0.75-1.14) | 0.476 |
| Haplotype3: | G | T | 90 | 164 | 0.94 (0.70-1.26) | 0.663 |
|  |  |  |  |  |  |  |
