# Supplementary material for: Bitter Taste Receptor Polymorphisms and Human Aging
Source: PLoS One. 2012 Nov 2;7(11):e45232. doi: 10.1371/journal.pone.0045232 (PMC3487725; doi:10.1371/journal.pone.0045232)
Supplement: Table S4 — Logistic regression analysis for haplotypes of T2R3-T2R4-T2R5- genes in long lived subjects. (DOCX) [file pone.0045232.s004.docx]

**Supplementary table S4: Logistic Analysis for Haplotypes of *T2R3-T2R4-T2R5-*genes in long lived subjects**

**Chromosome 7**

|  | **rs11763979** | **rs2233998** | **rs2234001** | **rs2227264** | |  |  |  |
| --- | --- | --- | --- | --- | --- | --- | --- | --- |
| **Haplotypes** | ***T2R3*** | ***T2R4*** | ***T2R4*** | ***T2R5*** | **≥85yrs^a^** | **<85yrs^a^** | **OR (95% CI)^b^** | **P_value_** |
| Haplotypes1: | T | C | C | T | 358 | 599 | 1 |  |
| Haplotypes2: | G | T | G | G | 285 | 562 | 0.86 (0.71-1.04) | 0.128 |
| Rare Haplotypes: | G | T | G | T | 5 | 13 | 0.64 (0.22-1.81) | 0.396 |
|  | G | T | C | G |  |  |  |  |
|  | G | C | C | T |  |  |  |  |
|  | T | T | G | G |  |  |  |  |
|  | T | T | C | G |  |  |  |  |
|  | T | C | C | G |  |  |  |  |
|  |  |  |  |  |  |  |  |  |
