# Supplementary material for: Bitter Taste Receptor Polymorphisms and Human Aging
Source: PLoS One. 2012 Nov 2;7(11):e45232. doi: 10.1371/journal.pone.0045232 (PMC3487725; doi:10.1371/journal.pone.0045232)
Supplement: Table S5 — Logistic regression analysis for haplotypes of TAS2R16 gene in long lived subjects. (DOCX) [file pone.0045232.s005.docx]

**Supplementary table S5: Logistic Analysis for Haplotypes of *TAS2R16* gene in long lived subjects**

|  | **rs1357949** | **rs6466849** | **rs860170** | **rs978739** | |  |  |  |
| --- | --- | --- | --- | --- | --- | --- | --- | --- |
| **Haplotypes** | ***T2R16*** | ***T2R16*** | ***T2R16*** | ***T2R16*** | **≥85yrs^a^** | **<85yrs^a^** | **OR (95% CI) ^b^** | **P_value_** |
| Haplotype1: | C | G | A | A | 218 | 390 | 1 |  |
| Haplotype2: | T | G | G | A | 233 | 357 | 1.17 (0.92-1.47) | 0.199 |
| Haplotype3: | T | A | A | G | 107 | 264 | 0.74 (0.56-0.98) | **0.033** |
| Haplotype4: | T | G | A | G | 69 | 134 | 0.93 (0.67-1.30) | 0.677 |
| Haplotype5: | T | G | A | A | 21 | 27 | 1.37 (0.75-2.48) | 0.303 |
|  |  |  |  |  |  |  |  |  |
