# Supplementary material for: Bitter Taste Receptor Polymorphisms and Human Aging
Source: PLoS One. 2012 Nov 2;7(11):e45232. doi: 10.1371/journal.pone.0045232 (PMC3487725; doi:10.1371/journal.pone.0045232)
Supplement: Table S6 — Logistic regression analysis for haplotypes of T2R38 gene in long lived subjects. (DOCX) [file pone.0045232.s006.docx]

**Supplementary table S6: Logistic Analysis for Haplotypes of *T2R38* gene in long lived subjects**

|  | **rs10246939** | **rs1726866** | **rs713598** | |  |  |  |
| --- | --- | --- | --- | --- | --- | --- | --- |
| **Haplotypes** | ***T2R38*** | ***T2R38*** | ***T2R38*** | **≥85yrs^a^** | **<85yrs^a^** | **OR (95% CI)^b^** | **P_value_** |
| Haplotype1: | C | G | C | 331 | 585 | 1 |  |
| Haplotype 2: | T | A | G | 290 | 556 | 0.92 (0.76-1.12) | 0.401 |
| Haplotype 3: | C | G | G | 26 | 31 | 1.50 (0.88-2.58) | 0.139 |
| Rare Haplotypes: | C | A | G | 1 | 2 | 0.94 (0.08-10.44) | 0.957 |
|  | T | G | G |  |  |  |  |
|  |  |  |  |  |  |  |  |
