# Supplementary material for: Bitter Taste Receptor Polymorphisms and Human Aging
Source: PLoS One. 2012 Nov 2;7(11):e45232. doi: 10.1371/journal.pone.0045232 (PMC3487725; doi:10.1371/journal.pone.0045232)
Supplement: Table S7 — Logistic regression analysis for haplotypes of T2R40 gene in long lived subjects. (DOCX) [file pone.0045232.s007.docx]

**Supplementary table S7: Logistic Analysis for Haplotypes of *T2R40* gene in long lived subjects**

|  | **rs10260248** | **rs534126** | |  |  |  |
| --- | --- | --- | --- | --- | --- | --- |
| **Haplotypes** | ***T2R40*** | ***T2R40*** | **≥85yrs^a^** | **<85yrs^a^** | **OR (95% CI)^b^** | **P_value_** |
| Haplotype1: | C | C | 348 | 670 | 1 |  |
| Haplotype2: | C | T | 241 | 415 | 1.13 (0.92-1.38) | 0.255 |
| Haplotype3: | A | T | 59 | 89 | 1.27 (0.89-1.81) | 0.185 |
|  |  |  |  |  |  |  |
