# Supplementary material for: Bitter Taste Receptor Polymorphisms and Human Aging
Source: PLoS One. 2012 Nov 2;7(11):e45232. doi: 10.1371/journal.pone.0045232 (PMC3487725; doi:10.1371/journal.pone.0045232)
Supplement: Table S8 — Logistic regression analysis for haplotypes of T2R41 in long lived subjects. (DOCX) [file pone.0045232.s008.docx]

**Supplementary table S8: Logistic Analysis for Haplotypes of *T2R41* in long lived subjects**

|  | **rs1404635** | **rs10278721** |  |  |  |  |
| --- | --- | --- | --- | --- | --- | --- |
| **Haplotypes** | ***T2R41*** | ***T2R41*** | **≥85yrs^a^** | **<85yrs^a^** | **OR (95% CI)^b^** | **P_value_** |
| Haplotype1: | G | C | 505 | 926 | 1 |  |
| Haplotype2: | A | T | 142 | 248 | 1.05 (0.83-1.32) | 0.711 |
|  |  |  |  |  |  |  |
