# Supplementary material for: Bitter Taste Receptor Polymorphisms and Human Aging
Source: PLoS One. 2012 Nov 2;7(11):e45232. doi: 10.1371/journal.pone.0045232 (PMC3487725; doi:10.1371/journal.pone.0045232)
Supplement: Table S9 — Logistic regression analysis for haplotypes of T2R7 -T2R9 genes in long lived subjects. (DOCX) [file pone.0045232.s009.docx]

**Supplementary table S9: Logistic Analysis for Haplotypes of *T2R7 -T2R9* genes in long lived subjects**

**Chromosome 12**

|  | **rs2588350** | **rs619381** | **rs3741845** | |  |  |  |
| --- | --- | --- | --- | --- | --- | --- | --- |
| **Haplotypes** | ***T2R7*** | ***T2R9*** | ***T2R9*** | **≥85yrs^a^** | **<85yrs^a^** | **OR (95% CI)^b^** | **P_value_** |
| Haplotype1: | G | G | C | 426 | 789 | 1 |  |
| Haplotype2: | G | G | T | 103 | 143 | 1.35 (1.02-1.78) | **0.037** |
| Haplotype3: | A | A | T | 79 | 152 | 0.99 (0.73-1.33) | 0.921 |
| Haplotype4: | A | G | T | 52 | 79 | 1.24 (0.86-1.80) | 0.255 |
| Rare Haplotypes: | G | A | T | 2 | 5 | 0.69 (0.13-3.61) | 0.664 |
|  | A | G | C |  |  |  |  |
|  | A | A | C |  |  |  |  |
|  |  |  |  |  |  |  |  |
