# Supplementary material for: Bitter Taste Receptor Polymorphisms and Human Aging
Source: PLoS One. 2012 Nov 2;7(11):e45232. doi: 10.1371/journal.pone.0045232 (PMC3487725; doi:10.1371/journal.pone.0045232)
Supplement: Table S10 — Logistic regression analysis for haplotypes of T2R14-T2R50-T2R20 genes in long lived subjects. (DOCX) [file pone.0045232.s010.docx]

**Supplementary table S10: Logistic Analysis for Haplotypes of *T2R14-T2R50-T2R49* genes in long lived subjects**

|  | **rs3916060** | **rs10772397** | **rs1376251** | **rs6488334** | **rs7135018** | **rs7301234** |  |  |  |  |
| --- | --- | --- | --- | --- | --- | --- | --- | --- | --- | --- |
| **Haplotypes** | ***T2R14*** | ***T2R50*** | ***T2R50*** | ***T2R50*** | ***T2R49*** | ***T2R49*** | **≥85yrs^a^** | **<85yrs^a^** | **OR (95% CI)^b^** | **P_value_** |
| Haplotype1: | T | A | T | G | C | T | 258 | 459 | 1 |  |
| Haplotype2: | T | G | C | A | T | C | 117 | 185 | 1.14 (0.87-1.51) | 0.345 |
| Haplotype3: | T | G | C | G | C | C | 95 | 189 | 0.92 (0.69-1.23) | 0.567 |
| Haplotype4: | T | A | C | G | C | C | 101 | 172 | 1.05 (0.78-1.40) | 0.764 |
| Haplotype5: | C | A | T | G | C | T | 61 | 102 | 1.06 (0.75-1.51) | 0.744 |
| Haplotype6: | T | A | C | G | C | T | 20 | 31 | 1.18 (0.66-2.12) | 0.575 |
| Haplotype7: | T | G | C | A | C | C | 10 | 27 | 0.67 (0.32-1.41) | 0.288 |
|  |  |  |  |  |  |  |  |  |  |  |
