# Supplementary material for: Bitter Taste Receptor Polymorphisms and Human Aging
Source: PLoS One. 2012 Nov 2;7(11):e45232. doi: 10.1371/journal.pone.0045232 (PMC3487725; doi:10.1371/journal.pone.0045232)
Supplement: Table S11 — Logistic regression analysis for haplotypes of T2R19-T2R31-T2R46-T2R30 genes in long lived subjects. (DOCX) [file pone.0045232.s011.docx]

**Supplementary table S11: Logistic Analysis for Haplotypes of *T2R48-T2R44-T2R46-T2R47* genes in long lived subjects**

|  | **rs10772420** | **rs10845293** | **rs12370363** | **rs10845296** | **rs2708381** | **rs2708380** | **rs2599404** |  |  |  |  |
| --- | --- | --- | --- | --- | --- | --- | --- | --- | --- | --- | --- |
| **Haplotypes** | ***T2R48*** | **T2R44** | **T2R44** | **T2R44** | **T2R46** | **T2R46** | **T2R47** | **≥85yrs^a^** | **<85yrs^a^** | **OR (95% CI)^b^** | **P_value_** |
| Haplotype1: | C | C | T | T | G | T | T | 262 | 471 |  |  |
| Haplotype2: | C | C | T | C | G | T | T | 154 | 300 | 0.94 (0.73-1.20) | 0.599 |
| Haplotype3: | T | T | C | T | A | A | G | 117 | 186 | 1.15 (0.87-1.52) | 0.324 |
| Haplotype4: | T | T | T | T | G | A | G | 102 | 170 | 1.08 (0.81-1.44) | 0.606 |
| Haplotype5: | T | T | T | T | G | T | G | 19 | 33 | 1.06 (0.59-1.91) | 0.845 |
| Rare Haplotypes | C | C | T | C | G | A | G | 8 | 8 | 1.75 (0.65-4.73) | 0.271 |
|  | C | T | C | T | A | A | T |  |  |  |  |
|  | T | T | C | T | G | A | G |  |  |  |  |
|  | C | C | T | T | G | T | G |  |  |  |  |
|  | C | C | T | T | G | A | T |  |  |  |  |
|  | C | C | T | C | G | T | G |  |  |  |  |
|  | C | C | T | C | G | A | T |  |  |  |  |
|  | C | C | T | C | A | T | T |  |  |  |  |
|  | T | T | C | T | G | T | T |  |  |  |  |
|  | T | C | T | C | G | T | T |  |  |  |  |
|  |  |  |  |  |  |  |  |  |  |  |  |

**^a^** Numbers may not add up to 100% of subjects due to genotyping failure. Data points that were still not filled after this procedure were left blank.

**^b^** OR: odds ratio; CI: confidence interval
